# Therapeutic disruption of *Plasmodium vivax* infected red cell deformability

**DOI:** 10.1186/1475-2875-13-S1-O25

**Published:** 2014-09-22

**Authors:** Rou Zhang, Wenn-Chyau Lee, Benoit Malleret, Rossarin Suwanarusk, Ming Dao, Cindy Chu, Chwee Teck Lim, Laurent Renia, Francois Nosten, Bruce Russell

**Affiliations:** 1 Department of Microbiology, Yong Loo Lin School of Medicine, National University of Singapore, Singapore; 2 Department of Parasitology, Faculty of Medicine, University of Malaya, Kuala Lumpur, Malaysia; 3 Singapore Immunology Network (SIgN), A*STAR, Biopolis, Singapore; 4 Centre for Tropical Medicine, Nuffield Department of Medicine, University of Oxford, Oxford, UK; 5 Shoklo Malaria Research Unit, Mahidol-Oxford Tropical Medicine Research Unit, Faculty of Tropical Medicine, Mahidol University, Mae Sod, Thailand; 6 Department of Materials Science and Engineering, Massachusetts Institute of Technology, Cambridge, MA, USA; 7 Department of Biomedical Engineering Faculty of Engineering, National University of Singapore, Singapore

## Background

Chloroquine (CQ) and artesunate (AS) are widely used as blood schizontocides in *P. vivax* treatment. Recent clinical observations show late stage parasites are cleared more rapidly than expected post treatment. As the high deformability of *P. vivax* facilitates its escaping from the splenic clearance, we hypothesize that CQ and AS directly affect the *P. vivax* infected red blood cells (iRBCs) rigidity. As a consequence, parasites are rapidly cleared from the blood circulation.

## Materials and methods

*P. vivax* isolates from Thailand were pulse incubated with AS, CQ and a spiroindolone (NITD609). Morphological changes and rosetting frequency were assessed by sub vital staining. The micropipette aspiration technique was the used to quantify the cell membrane shear modulus. Microfluidics were used to study the *in vitro* iRBCs behaviour after drug treatment.

## Results

While CQ and AS did not directly affect iRBC shear modulus, it significantly enhanced rosetting frequency and consequently the rigidity of rosetted iRBCs (the attachment of a single red cell results in a significant increase in shear modulus of the iRBC). NITD609 directly affected the iRBC rigidity. A microfluidic model of the spleen shows that *P. vivax* iRBCs with a higher rigidity are removed from flow. This study also show that normocytes that rosette with *P. vivax* iRBCs; form strong attachments (~500pN) that withstand a range of physiological shear stresses.

## Conclusions

In addition to providing new and important baseline biomechanical data on *P. vivax* rosettes; this *ex vivo* study also provides a possible explanation for the clinically observed disappearance of *P. vivax* parasites soon after treatment.

